# Circular stripes were more common in Barrett’s esophagus after acetic acid staining

**DOI:** 10.1186/s12876-018-0745-7

**Published:** 2018-01-25

**Authors:** Yating Sun, Shiyang Ma, Li Fang, Jinhai Wang, Lei Dong

**Affiliations:** 1 Department of Gastroenterology, the Second Affiliated Hospital of Xi’an Jiaotong University, No. 157 Xiwu Road, Xi’an, Shaanxi 710004 China; 2 Endoscopy Center, Ankang People’s Hospital, Ankang, 401147 China

**Keywords:** Barrett’s esophagus, Chromoendoscopy, Esophagogastric junction, Intestinal metaplasia

## Abstract

**Background:**

The diagnosis of Barrett’s esophagus (BE) is disturbed by numerous factors, including correct gastroesophageal junction judgment, the initial location of the Z-line and the biopsy result above it. The acetic acid (AA) could help to diagnose BE better than high resolution imaging technology or magnifying endoscopy, by providing enhanced contrast of different epithelium. We have noticed AA could produce multiple white circular lines, forming circular stripes (CS), at lower esophagus, which hasn’t been reported by others. This study aimed to investigate whether the CS is a special marker in BE patients.

**Methods:**

A total of 47 BE patients and 63 healthy people were enrolled from March 2016 to October 2016, and 2% AA staining had been operated routinely at lower esophagus under high resolution gastroscopy. We observed whether there were CS after AA staining and the images were compared between the two groups.

**Results:**

CS were confirmed in 42 patients (89.36%) in the BE group and 5 (7.94) in the control group ((*χ*^2^ = 72.931, *P* < 0.001)). The average width of CS was 0.76 ± 0.25 cm in BE group, which was similar to that in the control group (0.88 ± 0.11 cm). Villous or punctate or reticular pattern usually existed above or below the CS.

**Conclusions:**

CS could be found at lower esophagus in most BE patients with AA staining, and this special feature might be valuable in diagnosing, evaluating and following up of BE patients.

**Electronic supplementary material:**

The online version of this article (10.1186/s12876-018-0745-7) contains supplementary material, which is available to authorized users.

## Background

Barrett’s esophagus (BE) is defined as the replacement of squamous epithelium of the lower esophagus by single layer columnar epithelium [1–4], with or without the intestinal metaplasia (IM), which may be accompanied by risk of progression to carcinoma [4–6]. In recent years, the morbidity of esophageal squamous cell carcinoma and gastric carcinoma has been decreasing, while the incidence of esophageal adenocarcinoma is gradually increasing [5]. Therefore BE has attracted more attention as the most important precancerous lesion of esophageal adenocarcinoma.

The diagnosis of BE is disturbed by numerous factors clinically. Firstly, judgment for esophagogastric junction (EGJ) is subjective to some extent [7]. EGJ is usually defined by the top of the gastric folds, or the location of esophagus palisade blood vessel [8–13], both of which will be influenced by respiration, the volume of gas injected, the pressure of esophagus, even the relative position of diaphragmatic hiatus [9]. Secondly, the shape of the Z-line is another disturbing factor, and the biopsy result of columnar epithelium is meaningless if the initial or correct location of Z-line is wrong. Therefore, the accurate diagnosis of BE is based on correct EGJ judgment, the initial location of the Z-line, and the biopsy result above the Z-Line.

High resolution imaging technology and magnifying endoscopy have greatly improved the observation of mucosal micro-pattern. However, chromoendoscopy is irreplaceable. The acetic acid (AA) could provide better contrast of different epithelium. It is a kind of dye that can react specifically and reversibly with the columnar cells, however the exact mechanism is unclear yet. It is speculated that reversible degeneration of cellular proteins causes aceto-whitening reaction [14]. There are studies confirming that the AA used for BE epithelial staining could identify the mucosal microstructure especially highlight dysplasia and early cancer [15–18]. Meanwhile AA can increase the contrast between the squamous and columnar epithelium, producing white line at the junction which is coincident with Z-line in the healthy people. We have noticed that AA could produce multiple white circular lines, forming circular stripes (CS) at lower esophagus, and this feature was more common in BE patients. So a retrospective study was conducted to investigate whether the CS is a special marker for BE patients.

## Methods

### Patients

Both BE patients and control group participants were selected from Data of Endoscopy Center of The 2nd Affiliated Hospital of Xi’an Jiaotong University from March 2016 to October 2016.

Inclusion criteria: The BE patients should be diagnosed as full range or tongue type at least once before the research with pathological evidenc according to biopsy standard of ACG Clinical Guideline**,** 18 to 85 years old, male or female, outpatient or hospitalization. In this research, there are also some typical endoscope features can be seen to support BE diagnose, including columnar epithelium above esophagogastric junction (EGJ) where should be squamous epithelium normally, repositioned Z-line (upward the normal position > 0.5 cm) and the orange esophageal epithelium below the Z-line which is carnation in healthy people, and paliform vessel below the Z-line can be observed. If without the imaging evidence above, the patient will be removed out from the study.

In the control group, healthy physical examination participants with similar age and sex were selected. Esophageal AA staining was performed in both groups during the routine gastroscopy procedure and the images were fully integrated to identify the epithelial structure of lower esophagus near the Z-line.

**Fig. 1:**
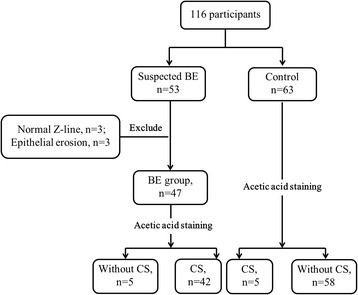
Flowchart of participants included

Exclusion criteria: Patients with esophageal epithelial erosion which is always lead by gastroesphageal reflux disease (GERD) and will impact the mucosa observation after AA staining (Additional file 1), patients with esophageal or gastric cancer, patients with acute gastrointestinal bleeding, and patients with upper gastrointestinal surgery were excluded. Inappropriate patients, such as normal Z-line location in BE group, or BE in control group, were excluded either (Fig. 1).

### Materials and equipments

AA was prepared as a 2% solution by diluting 5 mL of 6% acetic acid in (with) 10 mL of distilled water used for dyeing. High resolution gastroscopes (EG29-i10; PENTAX, Japan) were used for recording pictures and videos.

#### Protocol

The endoscope would be placed at the lower esophagus, or proximal end of the lesion if there was obvious metaplasia. The mucosa should be cleaned by injecting water before taking photos. Then 10 − 15 ml AA would be sprayed by spraying pipe onto cleaned mucosa, and further observation began after 30 s [19]. The biopsy were taken in and out of CS region with 1~ 2 pieces separately. Patients were given spasmolytic (Anisodamine 10 mg im.) and sedation (Midazolam 5 mg im.) before the examination to reduce the discomfort.

### Diagnostic criteria for CS

Made the Z-line and the submucosa of the esophageal folds evenly with moderate air under endoscope. The imaging features before AA staining were recorded. Thirty seconds after AA staining, the mucosa of lower esophagus and cardia turned white at the same time, which meaned the albino acetate reaction. Then, white linear stripes, so-called circular stripes, generally with a length of more than 0.5 cm, could be observed below the Z-line. These stripes had clear boundaries and distributed in circular or a certain quadrant. The images after AA staining were recorded. About 4–6 min later, the whitening area gradually returned to normal color and shape. This phenomenon is defined as CS positive in this study, which is approved and reviewed by two experienced endoscopic physicians.

All patients signed the informed consent of gastroscopy examination and AA staining. All aspects of the study were conducted using de-identified photographs and videos. Because all the photographs and videos existed before initiation of the study, this study was granted exempt status by the Xi’an Jiaotong University Human Research Committee.

#### Outcomes

The primary end point of the study was whether there were CS. The secondary outcome included the distribution and width of the CS, the length from the Z-line to the EGJ (presumed by CS) which was recorded using the Prague C&M criteria [20], the fine structure of the mucosa below the Z-line according to Guelrud M’s study [21],and the clinical symptoms of patients in BE group.

#### Statistical methods

All analyses were performed with SPSS 18.0 software. *χ*^2^ test or Fisher’s exact test were used to compare the categorical variables. As the age for each group was normally distributed and had equal variance, *t*-test was conducted to test their mean difference. Statistical difference was considered to be significant at the level of 0.05.

## Results

### The general characteristics of the subject

A total of 110 people were enrolled in the study and there were 47 patients in the BE group. Consistent with our patient population, the majority of the patients were over-aged with a roughly equal distribution between males and females (Table 1). In the BE group, the main clinical symptoms were not the same. 19 patients (40.42%) had acid reflux and heartburn, 12 (25.53%) had upper abdominal pain, 7 (14.89%) had abdominal distention, 5 (10.64%) had abdominal discomfort, and 4 (8.51%) had no typical symptom. In control group, there was no patient having symptom, consistent with the healthy screening population. There were not significant differences between two group of their taste preference (Table 1).

**Table 1:** Baseline characteristics of the two groups

	BE group *n* = 47	Control group *n* = 63	χ^2^	*P* value
Age, mean ± SD	53.68 ± 14.39	49.41 ± 11.51	1.728^a^	0.087
Female, n (%)	16 (34.0)	28 (44.4)	1.214	0.271
Fissure hernia, n (%)	6 (12.8)	4 (6.3)	1.341	0.320^*^
Taste preference				
Peppery	14 (29.79)	21 (33.33)	5.283	0.152
Sweet	11 (23.40)	18 (28.57)		
Sour	7 (14.89)	15 (22.22)		
Plain food	15 (31.91)	9 (14.29)		
Sympotoms (%)				
Acid reflux or heartburn	19 (40.42)	0		
Upper abdominal pain	12 (25.53)	0		
Abdominal distention	7 (14.89)	0		
Abdominal discomfort	5 (10.64)	0		
Asymptomatic	4 (8.51)	63 (100.00)		

^*^Fisher’s exact test

^a^t value from t-test

### Outcomes

In the BE group, the average M length of BE epithelium was 1.35 ± 0.48 cm (Prague criteria), and the C length was 0.50 ± 0.32 cm (Table 2). There was no long segment BE patient, and there were 38 (80.85%) patients with 1 cm ≤ M < 3 cm and 9 (19.14%) patients with M < 1 cm respectively. After acetic acid staining, CS was showed in a total of 42 patients (89.36%) in the BE group, which was significantly higher than that in the control group (5/63, 7.94%). There was a significant difference between the two groups (*χ*^2^ = 72.931, *P* < 0.001). CS could be found in the control group, which indicating movement of Z-line in 5 cases. The average width of CS was 0.73 ± 0.25 cm in the BE group, which was similar with that in the control group (0.88 ± 0.11 cm, *t* = − 1.270, *P* = 0.211).

**Table 2:** Results of gastroscopy and pathology in BE group vs control group

	BE group *n* = 47	Control group *n* = 63	χ^2^ or t value	*P* value
M value(cm), mean ± SD	1.35 ± 0.48	–		
C value(cm), mean ± SD	0.50 ± 0.32	–		
CS below the Z-line, n(%)	42 (89.36)	5 (7.94)	72.931	< 0.001
Width of CS (cm), mean ± SD	0.73 ± 0.25	0.88 ± 0.11	-1.270^a^	0.211
Above the CS				
Punctate pattern, n (%)	14 (33.33)	1 (20.00)	–	*
Reticular pattern, n (%)	13 (30.95)	3 (60.00)	–	*
Villous pattern, n (%)	14 (33.33)	0 (0.00)	–	*
Below the CS				
Punctate pattern, n (%)	19 (45.24)	2 (40.00)	–	*
Reticular pattern, n (%)	21 (50.00)	2 (40.00)	–	*
Villous pattern, n (%)	0	0	–	*
Without the CS	5 (10.6)	58 (92.1)	72.931	< 0.001
Punctate pattern, n	2 (40.00)	1 (1.72)	–	*
Reticular pattern, n	2 (40.00)	0 (0.00)	–	*
Villous pattern, n	1 (20.00)	0 (0.00)	–	*
Pathology confirmed intestinal metaplasia (IM), n (%)	23 (48.94)	3 (4.76)	29.101	< 0.001
The region of CS, n (%)	8 (34.78)	2 (66.67)	–	*
Above the CS, n (%)	13 (56.52)	1 (33.33)	–	*
Below the CS, n (%)	2 (8.70)	0 (0.00)	–	*

*The sample size of the variable is too small to do the hypothesis testing

^a^t value from t-test

**Fig. 2:**
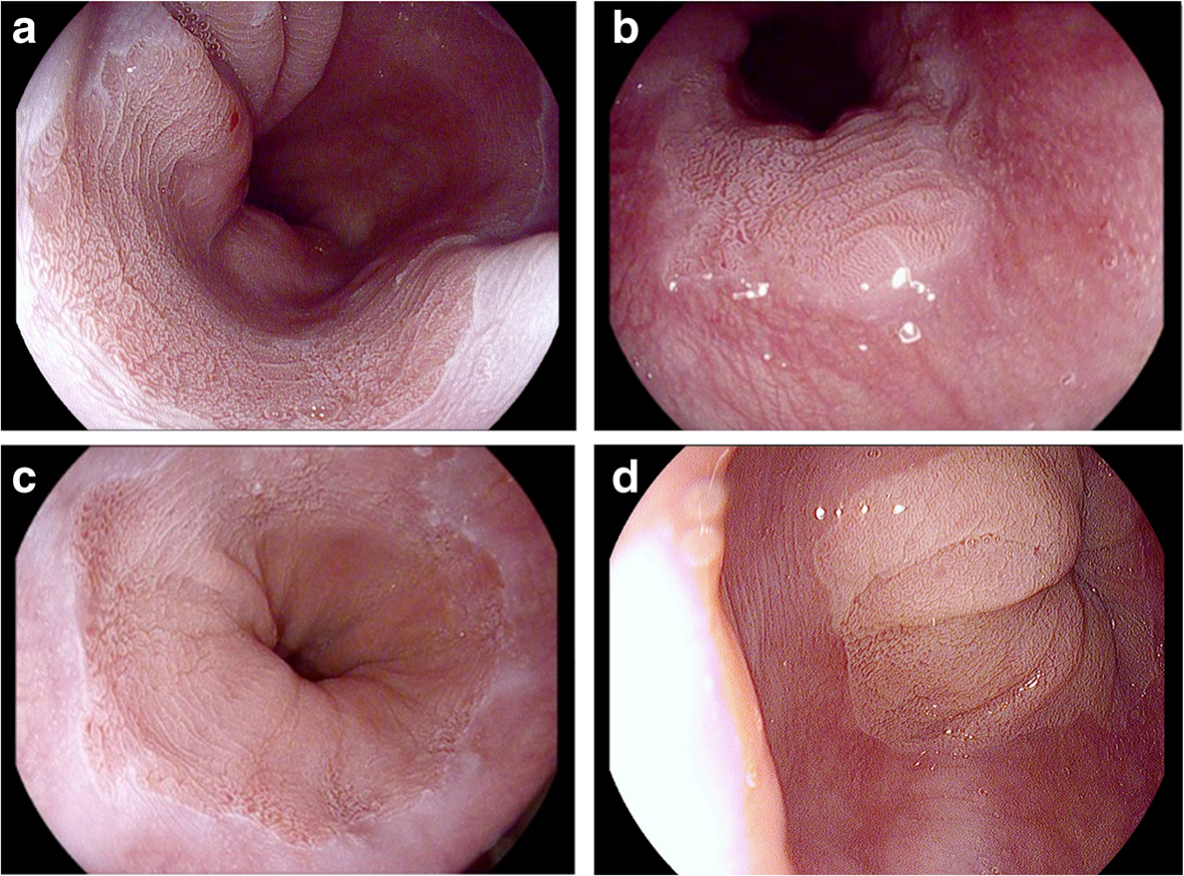
Aceto-whitening reaction for the diagnosis of BE after instillation of 2% acetic acid. **a**, **b**, **c** After spraying acetic acid, the mucosal surface shows multiple CS near EGJ, with the surface pattern could be identified by either reticular or punctate or villous. **d** normal mucosa with punctate pattern without CS

In BE group, mucosa patterns were always abnormal above the CS, including 33.33% villous (Fig. 2a), 30.95% reticular and 33.33% punctate pattern. Blow the CS, the reticular (50.00%) and punctate patterns (45.24%) were observed after staining (Fig. 2b, c). There were some abnormal mucosa patterns observed in control group (Table 2).

Without CS, the mucosa patterns were always normal, just 1 participant in control group without CS (1/58) was observed to have punctate pattern (Fig. 2d). But in BE group, although 5 patients didn’t have CS, their mucosa patterns were all abnormal.

Pathological examination showed that 23 (48.94%) patients had intestinal metaplasia (IM), which was significant more than control group (3/63, 4.76%). The patients with IM all had CS meanwhile. In BE group, 34.78% IMs were detected in the region of CS, 56.52% were above the CS and just 8.70% were below the CS.

## Discussion

Because the AA could give a good enhancement on the mucosa pattern at lower esophagus, we were using AA staining as a routine procedure for gastroscopy and the CS were unexpected discovery. In this study, CS were mostly observed in the BE group. There might be three potential mechanisms underneath this phenomenon.

**Fig. 3:**
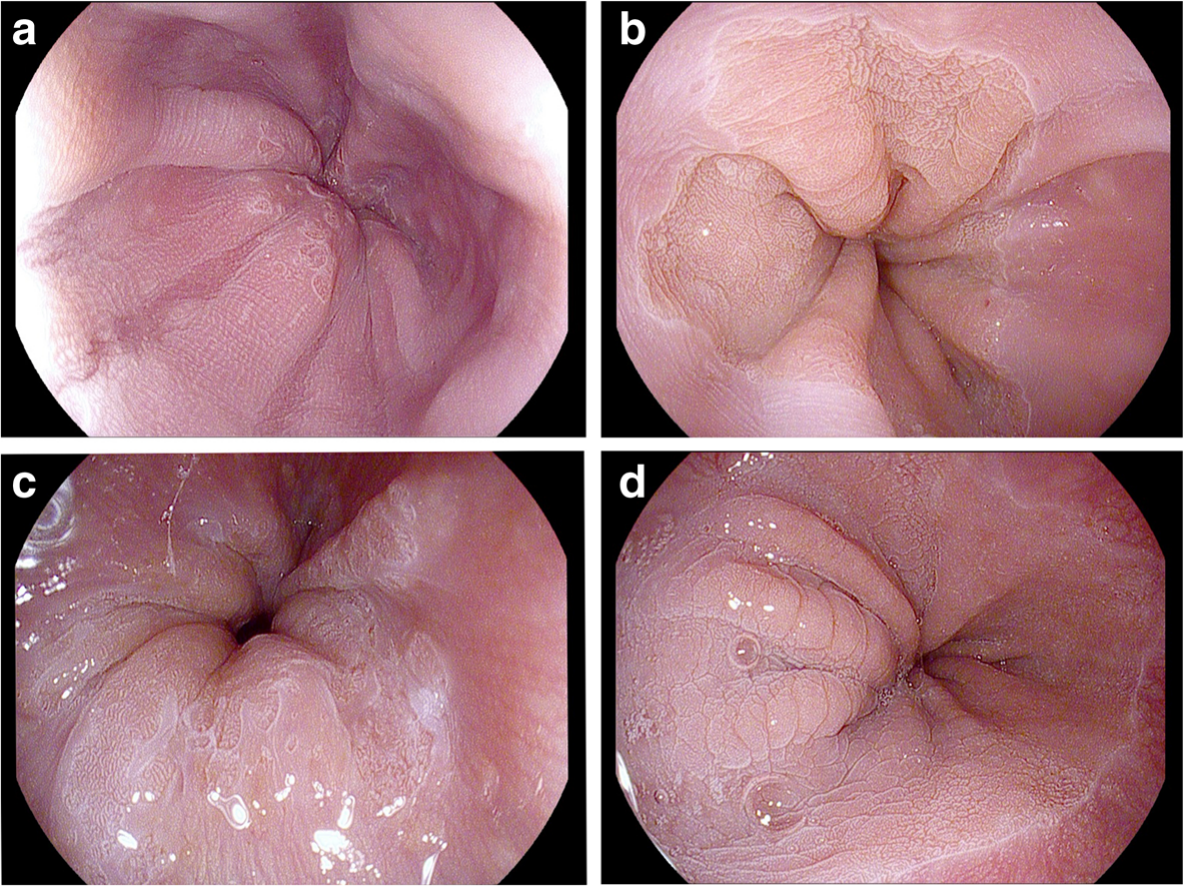
Special features after instillation of the 2% acetic acid. **a** Squamous epithelium was in circular pattern near the cardia in healthy people, **b** CS partially crossed from columnar epithelium to squamous epithelium, **c** Absence of CS in 2 BE patients and only villous or punctuate mucosa pattern were observed, **d** Flat circular appearance was observed in healthy people

Firstly, the CS might be caused by columnar epithelial metaplasia following squamous epithelium retraction. The AA might emphasize the gap between columnar epithelium in different periods. It could be found that the squamous epithelium was in circular pattern near the cardia in healthy people and the CS partially crossed from columnar epithelium to squamous epithelium (Fig. 3a, b), both supporting this explanation. However, the CS were only confined at the cardia within 2 cm. The emergence of new columnar epithelium in the higher position no longer formed CS anymore and could be found in punctate, reticular or villous pattern while the previous two mainly. On the other hand, there could be absence of CS in 5 BE patients and villous or punctuate mucosa pattern were observed (Fig. 3c). Therefore, the generation of the CS cannot be fully explained only by the regression and metaplasia theory.

The second possible explanation was that the CS might be specifically performed at the EGJ region and its scope exactly represented the range of EGJ. The hypothesis, that EGJ was not a simple line, but a small portion of the lower esophagus to the cardia, was suggested by previous studies [22–24]. The dense squamous epithelium covering the EGJ might be the reason why we could less likely observe CS in the control group. When the squamous epithelium gradually became thinner or replaced by columnar epithelium, the CS would be revealed. However, more evidence needs to be found.

Thirdly, the CS might be the result of repeated hyperplasia and substitution of the cardiac epithelium. The columnar epithelium of the cardia in BE patients might be affected by inflammation [25] or mechanical motion, which led to the edema or protein change of epithelium cell. For example, some healthy people were observed with circular appearance of the cardia in the inferior position occasionally. However, there was difference between non-metaplastic CS and metaplastic CS, that stripes in the former were flatter, while the latter were often stacked (Fig. 3d).

Based on the above assumptions, CS might help to identify the EGJ, sometimes obscure in white light images, even in magnified or NBI images. EGJ is an important marker for endoscopists to get biopsy, which is necessary for the diagnosis of BE in some guidelines, such as British Society of Gastroenterology guidelines and American Gastroenterological Association on the diagnosis of BE [1, 9]. Most studies suggested that EGJ was a marker for pointing the initial position of squamous epithelium, and further evaluating distance between the EGJ and the ascending squamo-columnar junction (SCJ) precisely [1, 9–11, 26]. Paris Workshop showed that the EGJ was located in the abdomen, just below the diaphragmatic pinch with the upper margin of the longitudinal gastric folds coinciding with the SCJ in the normal situation. The length of the metaplastic columnar segment is the distance between the neo-formed SCJ and the anatomical EGJ [26], and the reliability of the evaluation depends on the precision of the determination of the EGJ under endoscopy [26]. Mistake in EGJ judgement has little influence on diagnosis of long-segment BE, however it would mislead the diagnosis of short or ultra-short segment BE, while the majority of the Asian BE patients are in short segment [11, 26–31]. Ishimura showed that the prevalence of long segment BE was extremely low in East Asia, while the prevalence of short segment BE was very high only in Japan [11], which was similar to Okita K and Amano Y [30, 31]. Chang CY showed that short segment BE (75.6%, *n* = 31) was more prevalent than long segment BE (24.4%, *n* = 10) in Chinese population [28, 29]. Therefore, more effective method is needed to determine EGJ. If the CS after AA staining are related to the newly hyperplastic columnar epithelium, the length of the BE epithelium can be evaluated from distal end of CS; if CS are limited to the EGJ region, then the proximal end can be borderline for hyperplastic epithelium. Multiple biopsies may prolong the time of procedure and increase patients’ suffering, and cause too much bleeding to get the high-risk lesions [18, 32]. The CS helps to outline the target area and make emphasis on the microstructure of the surface. Meanwhile the fading effect after the AA staining can help to identify the abnormal mucosal lesions [14].

The research of the CS could also help to understand the origin and development of BE. Pathologists generally believe that the BE epithelium consists of three tissue types: [1] proximal end is intestinal epithelial cells including goblet cells, [2] in the middle, it is connection type epithelium that is cardiac mucosa without goblet cells, [3] the distal end is the basal epithelium contains both parietal cells and primary cells [33–36]. Our study indicates that CS may be a useful marker representing the connected epithelium perfectly and furthermore pathological evidences are required to support this theory.

It is generally believed that BE is closely associated with gastroesophageal reflux disease [1]. This study showed that BE patients mainly had gastroesophageal reflux symptoms, including acid reflux or heartburn, but 59.58% of patients had no symptoms of gastroesophageal reflux, which was similar to the literature reports [37–39]. These findings suggested that there may be other etiological factors, such as race, environment, diets, use of alcohol or smoking. Therefore, we should pay attention to the people without gastroesophageal reflux symptoms. Combined with magnifying endoscopy, Toyoda improved the mucosal microstructure classification through the study of patients with BE, including 3 types: normal pits, slit-reticular pattern, and gyrus-villous pattern. The sensitivity and specificity of gyrus-villous pattern for IM were 88.5% and 90.2%, and the overall accuracy was 90.0% [35]. In this study, we observed there was no IM in the punctate and the reticular area in the BE group, while the accuracy rate of IM was 100% in the villous area, which was consistent with Toyoda. This result suggested that AA staining combined with high resolution endoscopy could also improve the yielding of BE diagnosis without magnifying endoscopy, NBI or BLI, especially in screening.

The deficiency of this research was that the sample size was limited as a pilot study. In addition, we did not classify the different types of BE because of the small sample size. So further research is needed to explore the differences and the occurrence mechanism of different types of BE, and to explore the effectiveness of different endoscopic techniques in the diagnosis of BE epithelial range and nature.

## Conclusion

This is the pilot study that mentions and describes CS as a special feature under high resolution endoscopy with AA staining, and CS may become an important reference in the diagnosis and treatment of BE.

## Additional file

Additional file 1: The features after 2% acetic acid in patients with esophageal epithelial erosion. The esophageal epithelial erosion is always lead by gastroesphageal reflux disease and will impact the mucosa observation after acetic acid staining. (JPEG 666 kb)

## Abbreviations

AA: Acetic acid
BE: Barrett’s esophagus
CS: Circular stripes
EGJ: Esophagogastric junction
IM: Intestinal metaplasia
SCJ: Squamo-columnar junction

## References

[1] Fitzgerald RC, di Pietro M, Ragunath K (2014). British Society of Gastroenterology guidelines on the diagnosis and management of Barrett's oesophagus. Gut.

[2] Bennett C, Moayyedi P, Corley DA, et al. BOB CAT: a large-scale review and Delphi consensus for management of Barrett's esophagus with No Dysplasia, indefinite for, or low-grade Dysplasia. Am J Gastroenterol 2015; 110: 662–82 quiz 683. 10.1038/ajg.2015.55 PMC4436697 25869390

[3] Spechler SJ, Fitzgerald RC, Prasad GA, Wang KK (2010). History, molecular mechanisms, and endoscopic treatment of Barrett’s esophagus. Gastroenterology.

[4] Pereira AD, Chaves P (2016). Low risk of adenocarcinoma and high-grade dysplasia in patients with non-dysplastic Barrett's esophagus: results from a cohort from a country with low esophageal adenocarcinoma incidence. United European Gastroenterol J.

[5] Hvid-Jensen F, Pedersen L, Drewes AM, Sørensen HT, Funch-Jensen P (2011). Incidence of adenocarcinoma among patients with Barrett’s esophagus. N Engl J Med.

[6] van der Burgh A, Dees J, Hop WC, van Blankenstein M (1996). Oesophageal cancer is an uncommon cause of death in patients with Barrett’s oesophagus. Gut.

[7] Chandrasoma P, Makarewicz K, Wickramasinghe K, Ma Y, Demeester T (2006). A proposal for a new validated histological definition of the gastroesophageal junction. Hum Pathol.

[8] SA MC, Boyce HW, Gottfried MR (1987). Early diagnosis of columnar-lined esophagus: a new endoscopic criterion. Gastrointest Endosc.

[9] Spechler SJ, Sharma P, Souza RF, Inadomi JM, Shaheen NJ. American Gastroenterological Association technical review on the management of Barrett’s esophagus. Gastroenterology 2011;140:e18–e52; quiz e13. 10.1053/j.gastro.2011.01.031 PMC3258495 21376939

[10] Lee YC, Cook MB, Bhatia S (2010). Interobserver reliability in the endoscopic diagnosis and grading of Barrett’s esophagus: an Asian multi-national study. Endoscopy.

[11] Ishimura N, Amano Y, Sollano JD (2012). Questionnaire-based survey conducted in 2011 concerning endoscopic management of Barrett’s esophagus in east Asian countries. Digestion.

[12] Aida J, Vieth M, Ell C (2011). Palisade vessels as a new histologic marker of esophageal origin in ER specimens from columnar-lined esophagus. Am J Surg Pathol.

[13] Hoshihara Y (2000). Complications of gastroesophageal reflux disease. 2. Endoscopic diagnosis of Barrett esophagus—can Barrett esophagus be diagnosed by endoscopic observation alone?. Nihon Naika Gakkai Zasshi.

[14] Chedgy FG, Subramaniam S, Kandiah K, Thayalasekaran S, Bhandari P (2016). Acetic acid chromoendoscopy: improving neoplasia detection in Barrett's esophagus. World J Gastroenterol.

[15] Fortun PJ, Anagnostopoulos GK, Kaye P (2006). Acetic acid-enhanced magnification endoscopy in the diagnosis of specialized intestinal metaplasia, dysplasia and early cancer in Barrett's oesophagus. Aliment Pharmacol Ther.

[16] Lambert R, Rey JF, Sankaranarayanan R (2003). Magnification and chromoscopy with the acetic acid test. Endoscopy.

[17] Longcroft-Wheaton G, Brown J, Basford P, Cowlishaw D, Higgins B, Bhandari P (2013). Duration of acetowhitening as a novel objective tool for diagnosing high risk neoplasia in Barrett's esophagus: a prospective cohort trial. Endoscopy.

[18] Tholoor S, Bhattacharyya R, Tsagkournis O, Longcroft-Wheaton G, Acetic BP (2014). Acid chromoendoscopy in Barrett's esophagus surveillance is superior to the standardized random biopsy protocol: results from a large cohort study. Gastrointest Endosc.

[19] Kaufman HB, Harper DM (2004). Magnification and chromoscopy with the acetic acid test. Endoscopy.

[20] Sharma P, Dent J, Armstrong D (2006). The development and validation of an endoscopic grading system for Barrett's esophagus: the Prague C & M criteria. Gastroenterology.

[21] Guelrud M, Herrera I, Essenfeld H, Castro J (2001). Enhanced magnification endoscopy: a new technique to identify specialized intestinal metaplasia in Barrett’s esophagus. Gastrointest Endosc.

[22] Odze RD (2005). Unraveling the mystery of the gastroesophageal junction: a pathologist's perspective. Am J Gastroenterol.

[23] Pathology ORD (2005). Of the gastroesophageal junction. Semin Diagn Pathol.

[24] Wallner B (2009). Endoscopically defined gastroesophageal junction coincides with the anatomicalgastroesophageal junction. Surg Endosc.

[25] Savarino E, Marabotto E, Bodini G, et al. Epidemiology and natural history of gastro-esophageal reflux disease. Minerva Gastroenterol Dietol. 2017 Feb 17; 10.23736/S1121-421X.17.02383-2 28215067

[26] Paris workshop on columnar metaplasia in the esophagus and the esophagogastric junction, Paris, France, December 11–12, 2004. Endoscopy 2005;37:879–920. 10.1055/s-2005-870305 16116544

[27] Fock KM, Talley N, Goh KL (2016). Asia-Pacific consensus on the management of gastro-oesophageal reflux disease: an update focusing on refractory reflux disease and Barrett's oesophagus. Gut.

[28] Chang CY, Lee YC, Lee CT (2009). The application of Prague C and M criteria in the diagnosis of Barrett's esophagus in an ethnic Chinese population. Am J Gastroenterol.

[29] Tseng PH, Lee YC, Chiu HM (2008). Prevalence and clinical characteristics of Barrett's esophagus in a Chinese general population. J Clin Gastroenterol.

[30] Okita K, Amano Y, Takahashi Y (2008). Barrett's esophagus in Japanese patients: its prevalence, form, and elongation. J Gastroenterol.

[31] Amano A, Kinoshita Y (2008). Barrett esophagus: perspectives on its diagnosis and management in Asian populations. Gastroenterology & Hepatol.

[32] Bhattacharyya R, Longcroft-Wheaton G, Bhandari P (2015). The role of acetic acid in the management of Barrett's oesophagus. Clin Res Hepatol Gastroenterol.

[33] Bernstein IT, Kruse P, Andersen IB (1994). Barrett's oesophagus. Dig Dis.

[34] Toyoda H, Rubio C, Befrits R, Hamamoto N, Adachi Y, Jaramillo E (2004). Detection of intestinal metaplasia in distal esophagus and esophagogastric junction by enhanced-magnification endoscopy. Gastrointest Endosc.

[35] Paull A, Trier JS, Dalton MD, Camp RC, Loeb P, Goyal RK (1976). The histologic spectrum of Barrett's esophagus. N Engl J Med.

[36] Glickman JN, Spechler SJ, Souza RF, Lunsford T, Lee E, Odze RD (2009). Multilayered epithelium in mucosal biopsy specimens from the gastroesophageal junction region is a histologic marker of gastroesophageal reflux disease. Am J Surg Pathol.

[37] Chen X, Zhu LR, Hou KH (2009). The characteristics of Barrett's esophagus: an analysis of 4120 cases in China. Dis Esophagus.

[38] Park JJ, Kim JW, Kim HJ (2009). The prevalence of and risk factors for Barrett's esophagus in a Korean population: a nationwide multicenter prospective study. J Clin Gastroenterol.

[39] Lee IS, Choi SC, Shim KN (2010). Prevalence of Barrett's esophagus remains low in the Korean population: nationwide cross-sectional prospective multicenter study. Dig Dis Sci.

