# Supplementary figures and images for: Circular stripes were more common in Barrett’s esophagus after acetic acid staining
Source: BMC Gastroenterol. 2018 Jan 25;18:17. doi: 10.1186/s12876-018-0745-7 (PMC5784670; doi:10.1186/s12876-018-0745-7)

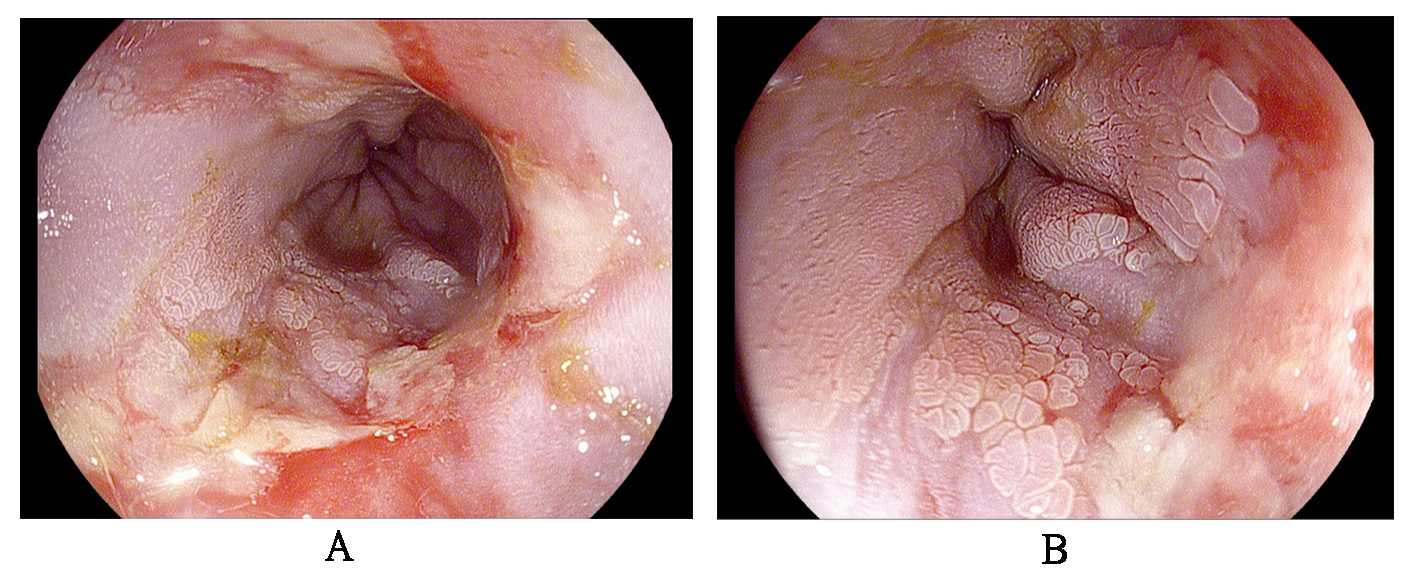

Supplement: Additional file 1: — The features after 2% acetic acid in patients with esophageal epithelial erosion. The esophageal epithelial erosion is always lead by gastroesphageal reflux disease and will impact the mucosa observation after acetic acid staining. (JPEG 666 kb) [file 12876_2018_745_MOESM1_ESM.jpg]
